# Spinal epidural lipomatosis presenting to a U.S. Veterans Affairs pain and rehabilitation department: a report of two cases

**DOI:** 10.1186/s12998-018-0203-1

**Published:** 2018-10-02

**Authors:** Keith M. Silcox, Clinton J. Daniels, Glenn A. Bub, Pamela J. Wakefield, James D. Toombs

**Affiliations:** 1 Syracuse VA Medical Center, Syracuse, NY USA; 2 Veterans Affairs Puget Sound Health Care System - American Lake, Tacoma, WA USA; 3 Adjunct Faculty Logan University, Chesterfield, MO USA; 4 Veterans Affairs Saint Louis Health Care System, St. Louis, MO USA; 5 Saint Louis University School of Medicine, St. Louis, MO USA

**Keywords:** Lipomatosis, Spinal stenosis, Chiropractic, Epidural steroid injection, Epidural fat, Case series

## Abstract

**Background:**

Spinal epidural lipomatosis is an uncommon source of neurogenic claudication. We present two cases of spinal epidural lipomatosis as it relates to diagnosis, management, and a possible association with common medical intervention.

**Case presentation:**

Case 1: 63-year old male patient presented with neurogenic claudication symptoms, but without evidence of bony central canal stenosis on lumbar computed tomography. He entered a trial of spinal manipulation with transient beneficial gains after seven appointments, but no durable change in neurogenic claudication. An MRI was recommended at this point which revealed grade III spinal epidural lipomatosis at the L5/S1 level.

Case 2: 51-year old male patient presented to a pain management physician with radicular symptoms for a series of lumbar epidural steroid injections. He completed a series of three lumbar epidural steroid injections with only short-term benefit. A repeat MRI demonstrated the presence of grade I (borderline grade II) spinal epidural lipomatosis.

**Conclusions:**

The first case illustrates a limitation of ruling out central canal stenosis with computed tomography for patients unable to undergo an MRI. The second case demonstrates a possible association between steroid injections and spinal epidural lipomatosis. An association of this kind has not been established; further research is needed to determine the significance.

## Background

Neurogenic claudication is the presentation of low back pain with bilateral or single leg pain, weakness, and/or paresthesia [1]. Claudication is categorically grouped into two types: vascular and neurogenic. Vascular claudication attributes symptoms to decreased perfusion of the lower extremities whereas neurogenic claudication is the result of stenosis within the central spinal canal. Stenosis may result for a number of reasons, but is typically found in an older population with bony degenerative changes [1]. The classic presentation of neurogenic claudication is pain with prolonged walking that is relieved while leaning forward, such as using a shopping cart, or sitting [1].

Another potential cause of central canal stenosis is spinal epidural lipomatosis (SEL). This is the accumulation of non-encapsulated adipose tissue in the epidural space. Borre et al. [2] developed a grading system to classify the amount of adipose tissue relative to the dural sac and spinal canal. This grading system took advantage of three measurements obtained by MRI: the total anterior to posterior diameter of the epidural fat (EF), the anterior to posterior diameter of the dural sac, and the anterior to posterior diameter of the spinal canal (Table 1) [2]. For the purposes of this case series the diameter of the spinal canal was measured in each image and either the dural sac or EF was measured with the third measurement found by subtracting the dural sac or EF from the spinal canal measurement.

**Table 1:** Created from the Borre et al. [2] classification system

	Grade I	Grade II	Grade III
Dural Sac Diameter ÷ Epidural Fat Diameter	1.49–1	0.99–0.34	≤0.33
Epidural Fat Diameter ÷ Spinal Canal Diameter	41–50%	51–74%	≥75%

The cause of adipose cell hypertrophy is unknown, however, the two most common characteristics found among previous case reports were long-term exogenous corticosteroid use and a body mass index (BMI), > 27.5 [3–9]. One case reported the development of SEL following a single lumbar epidural steroid injection [8]. Fogel et al. [4] reviewed 104 case studies and found that exogenous corticosteroid use accounted for 55% of cases, obesity 25%, idiopathic 17% and Cushing’s 3%. The objective of this report is to present two case studies as they relate to the diagnosis and management of spinal epidural lipomatosis as seen at the Veterans Affairs Saint Louis Health Care System.

## Case Presentation

### Case 1

A 63-year-old Caucasian male veteran was referred to the chiropractic clinic with a 2-year history of insidious, worsening low back and bilateral leg pain. The patient complained of pain and cramping in his lower legs that was provoked with walking and immediately relieved with sitting. He further described the left leg as mildly worse than the right. His walking was limited to approximately 50-ft due to pain, but leaning forward on a grocery cart greatly increased his capacity. His medication list included 81 mg aspirin once daily and short-term dose of hydrocodone/acetaminophen 30/300 mg for an unrelated condition (excision of a cervical sebaceous cyst). He was previously prescribed a trial of 300–900 mg Gabapentin, but discontinued without relief. His relevant medical history included right femur internal fixation for a traumatic intertrochanteric fracture, diabetes mellitus, open mandible surgery with hardware placement in the 1970’s, and left ankle surgery with temporary hardware in 1995. He did not have any history of anabolic or corticosteroid use, Cushing’s disease, or history of epidural steroid injections.

**Fig. 1:**
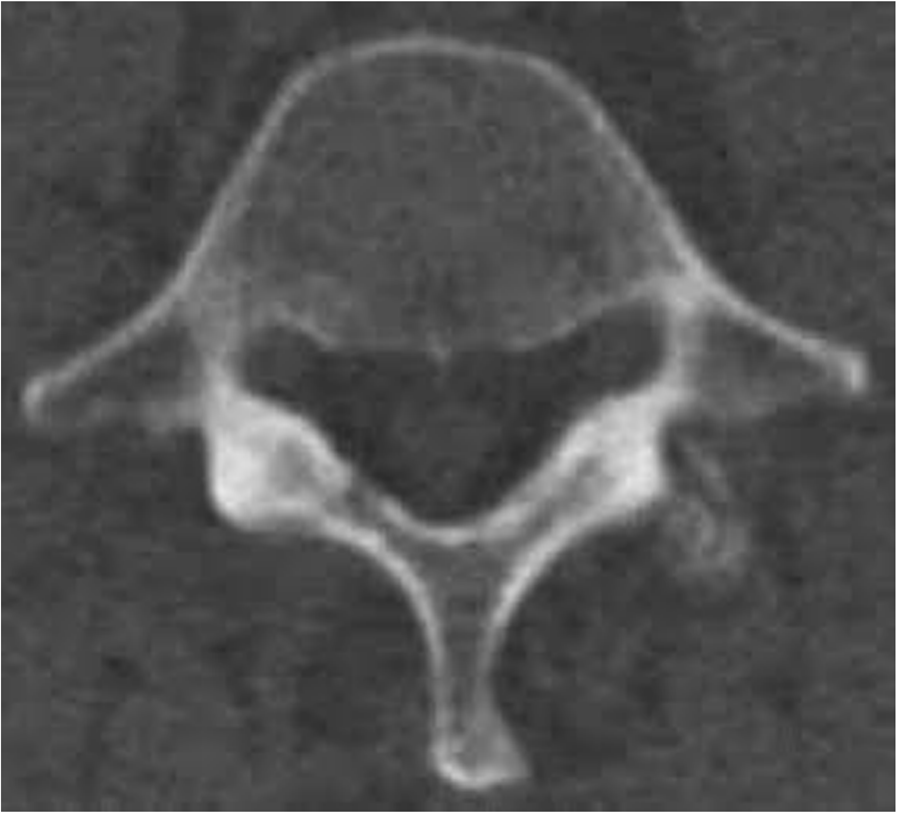
CT bone window image of L4–5 which does not clearly demonstrate evidence of spinal epidural lipomatosis

Relevant physical examination included a body mass index of 38. He reported weight gain in response to his pain, and chart notes corroborated a BMI of 32.5 approximately 2-years prior to presentation. The patient had moderate flexion and extension limitation with lumbosacral pain on extension. Sensory, motor and tendon reflexes were within normal limits. Lumbosacral pain was present with facet loading. Hip internal rotation and flexion limited bilaterally due to hamstring and hip musculature tightness. All other lumbar and pelvic orthopedic tests were unremarkable. On initial presentation, pain disability questionnaire [10] was scored as 63 out of 150, with a functional status component of 37 and a psychosocial component of 26. The patient presented with computed tomography; revealing suspected left foraminal disc protrusion at L4–5 and bilateral L4–5 and L5-S1 facet hypertrophy, but no bony spinal stenosis (Fig. 1).

He was diagnosed with neurogenic claudication and treated six times with flexion-distraction to the lumbar spine and high-velocity low amplitude manipulation to the lumbar and thoracic spine. Soft tissue manual therapy was performed on the hip external rotators bilaterally. He was instructed in repetitive lumbar end-range flexion (centralization phenomenon observed), hip mobility exercises, and sciatic nerve glides. At his seventh session a re-examination was performed. His hip flexion range of motion was improved, however he continued to experience pain with walking and cramping in his lower legs. Orthopedic testing was without significant change. His updated pain disability questionnaire scored 96/150, indicating a potential progression of his disability.

**Fig. 2:**
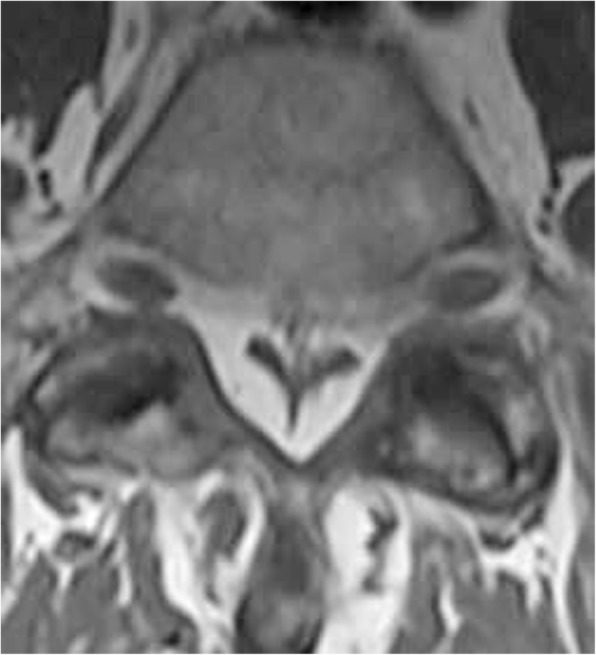
case 1, visualization of the “Y” sign indicating grade III compression

**Fig. 3:**
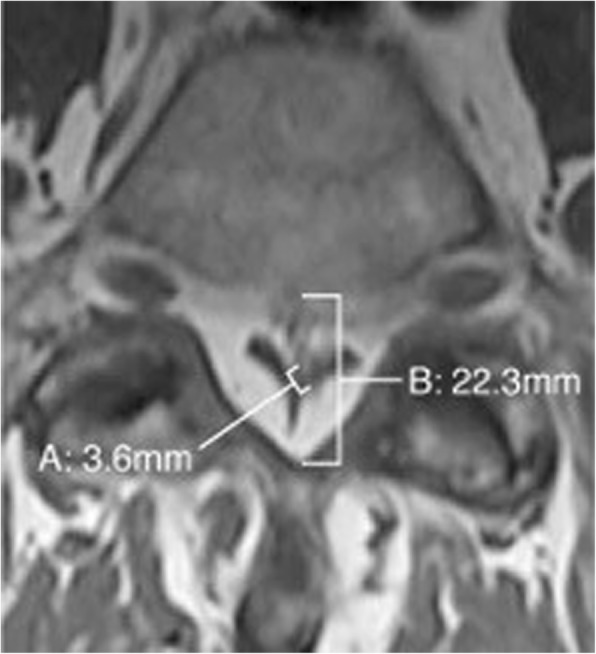
case 1, measurement of the “Y” sign using the modified method. “A” demonstrates the dural sac as measured with the modified method, “B” demonstrates the spinal canal, and EF is the difference between these measurements

At this time the patient was able to be cleared for a lumbar MRI which revealed no signs of bony or discogenic spinal stenosis; however circumferential epidural fat was present at L5-S1. Inspection of the patient’s T1 weighted MRI revealed the grade III pathognomonic “Y” sign (Fig. 2). A measurement of the patient’s epidural fat using the modified method (a method used when a straight anterior to posterior measurement is not possible) developed by Borre et al. [2] revealed the following: dural sac / epidural fat value of 0.19 and epidural fat / spinal canal value of 83.9% as measured by the authors (Fig. 3). This measurement also categorized the patient as a grade III. The patient was seen for two additional visits without any further durable gains and was referred by his medical provider for a neurosurgery consult. After meeting with the neurosurgeon, the patient opted not to pursue surgical intervention.

### Case 2

**Fig. 4:**
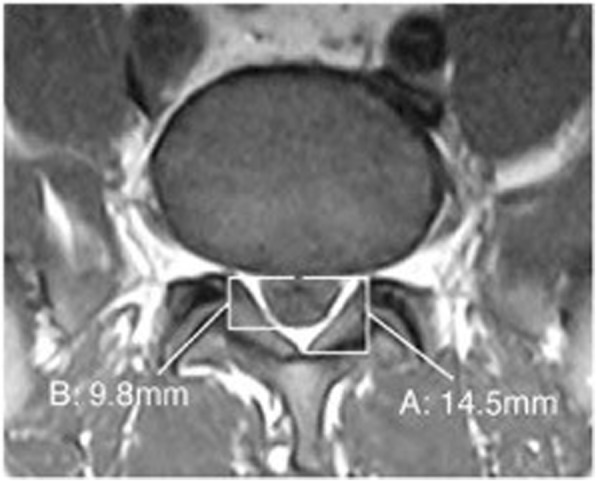
case 2, epidural fat measurement pre LESI, demonstrating a grade 0, or normal finding. “A” demonstrates the spinal canal, “B” demonstrates the dural sac, and EF is the difference between these measurements. The L4–5 disc herniation is not visualized on this image

**Fig. 5:**
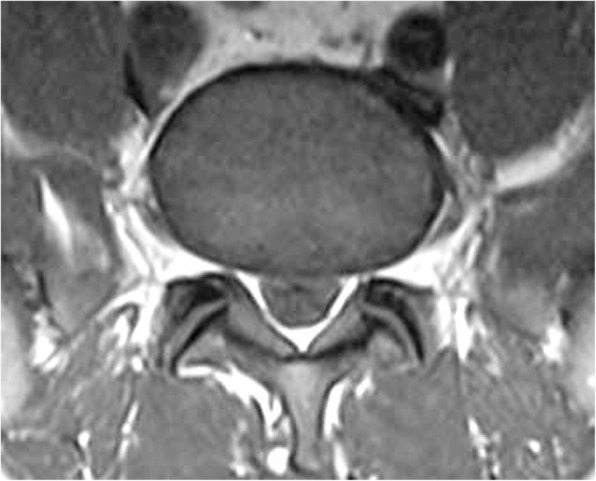
case 2, pre LESI imagine without measurements. The L4–5 disc herniation is not visualized on this image

A 51-year-old African American male veteran was referred to a pain management physician for a lumbar epidural steroid injection (LESI) for low back pain with radiculopathy. The patient had a history of low back pain and lower extremity pain for three and half years. Previous treatments included naproxen, arch supports, proper lifting education, and physical therapy. Radiographs demonstrated minimal spondylosis at the L4 and L5 vertebral bodies and a MRI demonstrated a mild posterior central disc herniation at L5-S1, but no evidence of SEL (Figs. 4 & 5).

The patient’s medical history included low back pain, hip pain, plantar fasciitis, obesity, and benign prostatic hyperplasia. The patient’s active medications included: meloxicam, terazosin HCL, ibuprofen, bisacodyl, cyclobenzaprine, methocarbamol, tramadol HCL, acetaminophen/hydrocodone, omeprazole, and ketorolac tromethamine. The patient had no history of anabolic or corticosteroid use or Cushing’s disease; BMI at the time of treatment was 34.

**Fig. 6:**
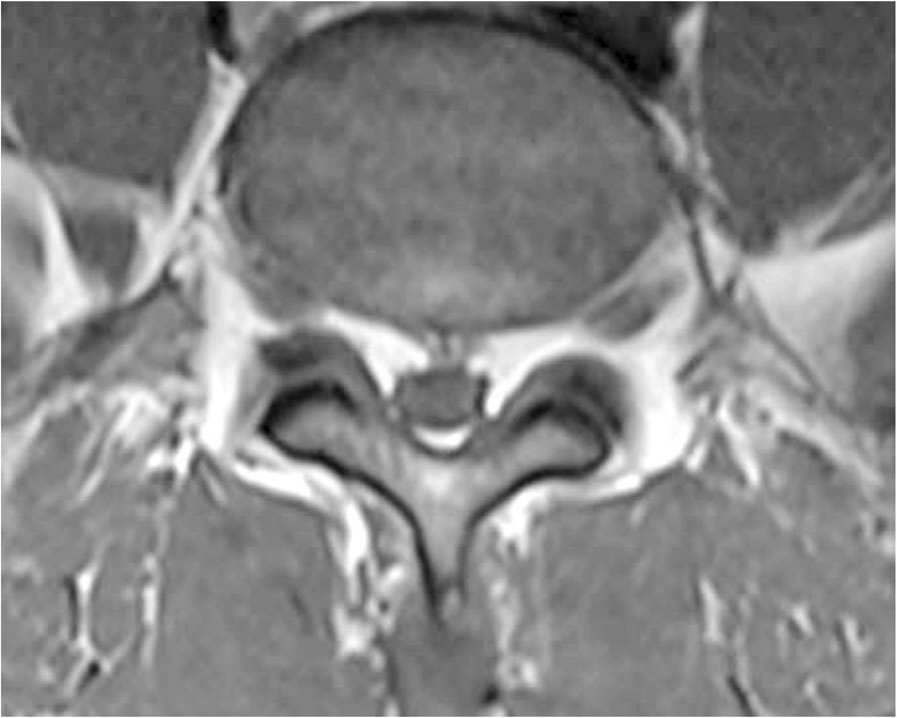
case 2, post LESI imagine without measurements

**Fig. 7:**
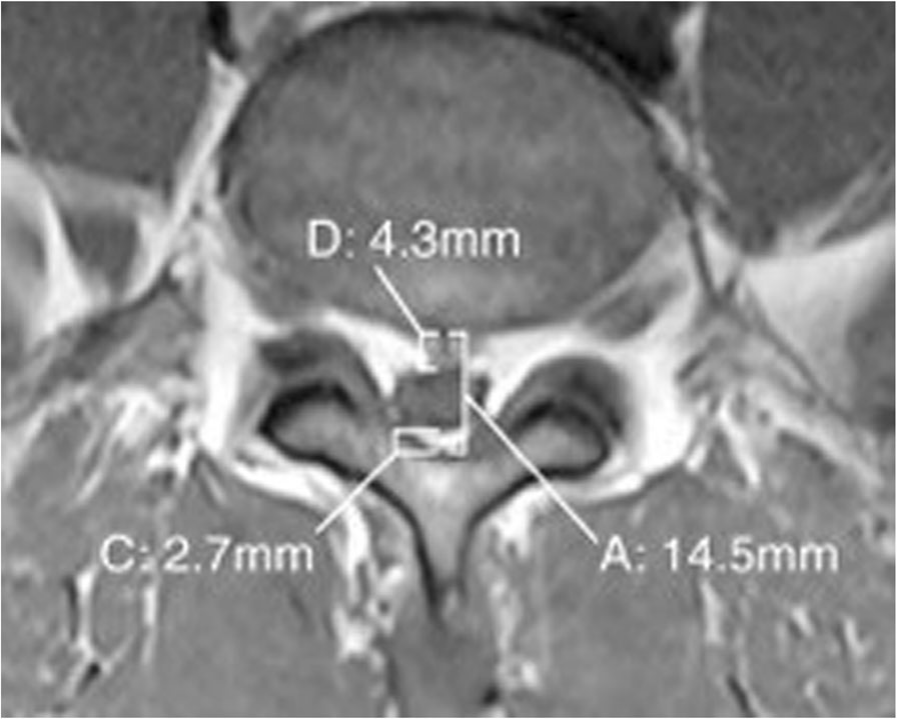
case 2, epidural fat measurement post LESI, demonstrating a grade I (borderline grade II) SEL. “A” demonstrates the spinal canal, “C” and “D” demonstrates the EF on either side of the dural sac, and the dural sac is the difference between A and C + D

Over a five-week period the patient received a series of three interlaminar L4–5 LESI. The patient reported short-term relief with each injection in the series. Three months after the final injection the patient was referred for a neurosurgery consult. The patient’s neurological exam was fully intact and a repeat MRI was ordered with the following impression: L2 level degenerative changes of the lumbar spine with epidural lipomatosis at lower lumbar levels which result in severe central canal stenosis at L4–5 and L5-S1 and varying degrees of neural foraminal narrowing. When measured by the authors using the method developed by Borre et al. [2], the dural sac diameter/epidural fat diameter was 1.07, and the epidural fat/spinal canal diameter was 48.3%, categorizing the patient as a grade I (borderline grade II) (Figs. 6 & 7). The initial MRI measured 2.09 (DS/EF) and 32.4% ((EF/SC) as measured by the authors, which results in a grade 0 categorization. It is important to note that in one review [2] only 14.5% of grade II cases, and 0% of grade I, were symptomatic. The time between the initial and repeat MRI was 5 months and the only two interventions during this time were a series of three LESI and the introduction of acetaminophen/hydrocodone. As a result of the repeat MRI findings surgical decompression was recommended.

## Discussion

Treatment options for neurogenic claudication include manipulation, active and passive physical therapies, medication, epidural steroid injections, and decompression surgery [1]. The evidence for chiropractic treatment options is sparse [1, 11–14]. However several studies have found that a combination of manual care methods, most often including flexion distraction and sciatic nerve mobilization, have been effective in reducing patient reported disability [11–14]. Ammendolia and Chow conducted a preliminary study incorporating a conservative multimodal treatment method with 49 patients, which demonstrated promising outcomes for neurogenic claudication [11]. It is unknown whether chiropractic care may be beneficial for neurogenic claudication that is the result of spinal epidural lipomatosis, as opposed to lumbar degeneration.

Borre et al. reviewed 2528 lumbar MRIs and found grade III SEL in 2.1% of patients, grade II in 6.5%, and grade I in 12.2% [2]. Their results identified that 100% of patients with a grade III, 14.5% of grade II, and 0% of grade I cases were symptomatic [2]. Sugaya et al. [3] specifically identified grade III patients and found the prevalence to be 0.33% of 1498 reviewed MRIs. All grade III cases identified by Sugaya et al. [3] were symptomatic. The classification system developed by Borre et al. [2] does not appear to be in widespread use. However, this method of grading SEL appears reproducible and would give clinicians a better understanding of symptoms related or not related to SEL. Additionally disease progression and treatments may be better monitored with a more universal use of this grading classification. The second case demonstrated a need for this universal grading method. When measured, this patient presented with a mild stenosis as opposed to the reported severe stenosis noted in the MRI report.

Previous studies [3–7, 9] have found an association between SEL and long-term prednisone use and SEL and obesity (≥ 27.5 BMI). The two patients in this study did not have a history of prednisone use or Cushing’s disease; however, both did have an elevated BMI at the time treatment was provided. It should be noted that elevated BMI was the only risk factor associated with 25% of cases seen by Fogel et al. [4] and was seen in 86.6% of the cases found by Borre et al. [2]. While the first case saw a weight elevation, possibly in response to pain related inactivity, it is likely that obesity played a role to some degree in both cases. The patients in both cases declined a weight loss program when offered.

It should be noted that of the cases with associated exogenous corticosteroid use or obesity a moderately successful treatment option was found in reducing the dose of corticosteroids or losing weight respectively [3, 4, 6, 7]. Although not found in the literature search, a repeat MRI to determine if these treatments reduced the grade of SEL would be clinically useful in helping to determine effectiveness of treatment and possibly in modifying diagnostic and treatment protocols. For those patients that continued to have symptoms surgical decompression was the most common treatment [3–7, 9]. Other commonly reported treatment options include manual care, NSAIDs, and epidural steroid injection [3–7, 9]. Rustom et al. [15] questioned the use of epidural steroid injections as a treatment due to failed demonstration of symptom relief and the possibility of accentuating adipose deposition.

A search of the literature revealed one previous case study with a possible association between a single steroid injection and SEL [8]. However, when this previous case study was examined further it became difficult to determine the significance of the increased epidural fat. Tok et al. [8] found an increase in epidural fat after a single LESI; however, they did not use the measuring classification developed by Borre et al. [2]. The authors did measure the epidural fat in the space ventral to the dural sac and found a 2.3 mm anterior posterior increase [8]. When the imagines in the Tok et al. [8] article are measured using the Borre et al. [2] method the patient in the case study was found to have a grade II SEL and a slightly worse, but still grade II SEL post injection. Since the SEL grade did not change it is difficult to determine if this result demonstrates a clinically significant change. If a significant change was determined to have taken place it would be difficult to rule out a natural progression of the patient’s already present grade II SEL.

The first case demonstrates the conservative management of a grade III symptomatic SEL case initially undiagnosed due to the limitations of using CT bone window images to assess for soft tissue changes within the spinal canal. The patient was referred for chiropractic care and presented with symptoms of low back pain with neurogenic claudication. A previous CT scan demonstrated no central canal stenosis to substantiate the physical exam findings of neurogenic claudication. An MRI was not initially ordered due to possible contraindications from old hardware in his mandible, but was indicated after the patient failed a trial of conservative care. This case demonstrated no long-lasting gains from chiropractic care for the treatment of grade III SEL. However, it did illustrate a potential shortcoming for patients with a negative CT scan, physical signs of neurogenic claudication, and contraindications for MRI. The patient in this case was ultimately able to be cleared for an MRI, which provided the diagnosis of SEL.

The second case seen here was a pain management case and demonstrated a possible association between LESI and the development of SEL. Elevated BMI was the only other associated risk factor the patient presented with for SEL. This case presented with an MRI demonstrating a normal amount of epidural fat followed by a repeated MRI 5 months later demonstrating a grade I, borderline grade II SEL. The two interventions seen during this time period were LESI and opioid pain medication. This one case does not indicate an association between LESI and SEL, especially given the patient’s elevated BMI, but it is notable since the patient presented with a grade 0 prior to LESI. More research should be performed to determine if an association exists.

## Limitations

The findings of these two cases are unique to these individuals and may not necessarily be extrapolated to the general population. Further, it is possible that SEL was an incidental finding on MRI and is not the cause of either patients’ symptoms. Additional limitations of the second case are that the patient’s symptoms do not appear to be consistent with classic symptoms of neurogenic claudication nor did the patient’s symptoms change significantly despite the additional SEL findings in the repeat MRI. However, additional symptoms would not be expected from the findings of a grade I SEL.

## Conclusion

Spinal epidural lipomatosis is an uncommon lumbar MRI finding ranging from 0.33–2.1% [2, 3]. A limitation of the diagnosis of this condition is found in patients contraindicated for MRI and cleared by CT scan. The pathogenic cause of SEL remains unknown, but appears that it is related to exogenous corticosteroid use and obesity. The findings in the second case of this study may warrant a closer look at the association between LESI and the development of SEL. Further research is needed to determine the exact cause of adipose cell hypertrophy, which would allow for a better understanding of patients at risk and lead to prevention strategies. Additionally, a more universal use of the grading classification seen here may lead to a better understanding of the associations, natural progression, and effective treatments for SEL.

## Abbreviations

BMI: Body mass index
CT: Computed tomography
DS: Dural sac diameter
EF: Epidural fat diameter
LESI: Lumbar epidural steroid injection
mg: Milligrams
MRI: Magnetic resonance imaging
NSAID: Non-steroidal anti-inflammatory drug
SC: Spinal canal
SEL: Spinal epidural lipomatosis
